# Stentectomy of a Balloon-Mounted Stent From Vertebrobasilar Circulation: A Technical Report and Literature Review

**DOI:** 10.7759/cureus.28956

**Published:** 2022-09-08

**Authors:** Sudheer Chakravarthi, Sai Kumar Reddy Pasya, Vamsi Krishna Gorijala, Anusha Guntamukkala, Kalyan Chakravarthy Sajja

**Affiliations:** 1 Department of Neurology, Life Neuro Vascular Institute, Guntur, IND; 2 Department of Endovascular Neurosurgery, Thomas Jefferson University, Philadelphia, USA

**Keywords:** neurointervention, ischemic stroke, dislodged stent, stent retrieval, stentectomy

## Abstract

Stents are being widely used in the neuroendovascular field more often for assisted coiling of aneurysms and treatment of atherosclerotic stenosis. Stent detachment and embolization are one of the most feared complications associated with poor clinical outcomes. Many techniques have been detailed in the literature for extracting such dislodged stents. We describe a case of retrieval of an inadvertently detached balloon-mounted stent from the intracranial left vertebral artery. This occurred in a 58-year-old male patient with a history of diabetes mellitus whose stenting procedure was planned for severe intracranial atherosclerotic disease of bilateral vertebral arteries causing recurrent posterior circulation ischemic events. Stentectomy was performed successfully using a stent retriever. Intracranial vertebral artery stenting was eventually accomplished with excellent clinical outcomes.

## Introduction

Complications associated with the usage of stents like stent dislodgement, herniation into the parent vessel, and stent fracture may be caused either due to the wrong selection of a device, inherent device malfunction [1], or due to anatomical factors such as tortuous arteries. With the increasing usage of different kinds of stents, interventionalists noticed a few device-related complications such as stent migration, misplacement, displacement, incomplete deployment, and foreign body embolization. Inadvertent stent detachment and embolization are one of the most feared complications associated with poor clinical outcomes. Leaving the dislocated stent in situ may result in hazardous complications such as traumatic injury to the vascular wall leading to perforation, local thrombogenicity, and ischemia. Hence, retrieval of misplaced stents should be attempted. Several techniques for stentectomy have been proposed in the literature with variable success ranging from surgical extraction to endovascular approaches using micro snares, stent retrievers, merci retrieval systems, balloon pullback maneuvers, deploy and engage technique/loop, and snare techniques [2-5]. Most of these stent retrieval techniques involving intracranial circulation were performed on self-expandable stents. Here, we report a case of inadvertent stent detachment and migration of a balloon-mounted stent in the vertebrobasilar segment that was salvaged using a stent retriever alone. To our knowledge, very few case reports were reported mentioning the use of a stent retriever alone to remove a detached balloon-mounted stent from the cerebral vasculature.

## Technical report

**Figure 1:**
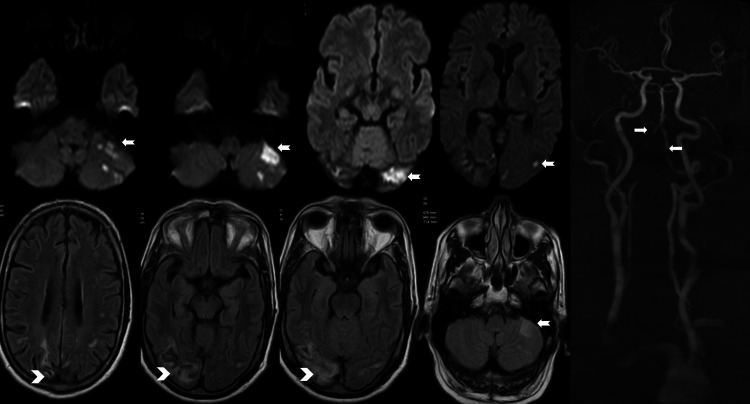
Evidence of severe stenosis was noted in the V3 and V4 segments of the right vertebral artery. Narrowed caliber V1 and V2 segments of the right vertebral artery from its origin. Stenosis was noted at the V4 segment of the left vertebral artery. Acute ischemic infarcts in the left cerebellar and left occipital region (notched arrows). Severe stenosis in the V4 segments of bilateral vertebral arteries on a magnetic resonance angiogram (arrows). Chronic ischemic changes in the right occipital and parietal region (arrowheads).

A 58-year-old male patient with a premorbid history of diabetes mellitus presented with sudden onset vertigo, gait ataxia, and blurring of vision. MRI of the brain revealed acute infarcts in the bilateral occipital region as well as in the bilateral cerebellar hemispheres. A magnetic resonance (MR) angiogram showed severe stenosis in the bilateral intracranial vertebral arteries in their V4 segment (Figure 1).

He was started on dual antiplatelets (aspirin 150 mg and clopidogrel 75 mg) along with statins and corrective insulin therapy. Three months later, he presented with another episode of acute vertigo along with transient left-sided weakness. CT of the brain revealed no acute insult to the brain and he was continued on dual antiplatelets. He continued to have recurrent episodes of dizziness and gait imbalance despite adequate medical management over a subsequent couple of months.

Cerebral digital subtraction angiography performed five months into his illness revealed occlusion of the right vertebral V4 segment, distal to the origin of the posterior inferior cerebellar artery (PICA) along with severe stenosis of the left vertebral artery V4 segment, close to the vertebrobasilar junction. Intracranial stenting of the left vertebral V4 segment stenosis was contemplated.

**Figure 2:**
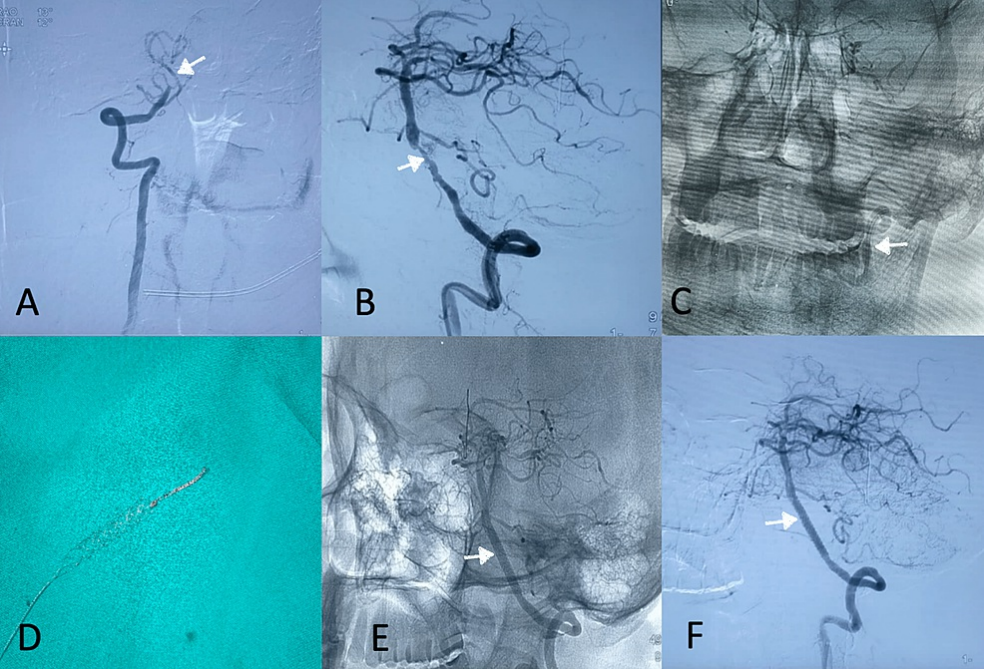
(A) Occlusion of the right vertebral artery V4 segment distal to the origin of PICA. (B) Severe stenosis in the left vertebral artery V4 segment proximal to the origin of the AICA-PICA complex. (C) Dislodged stent in the left vertebral artery V3 segment. (D) Dislodged stent captured by stent retriever. (E) The stent is seen in situ in the left vertebral V4 segment (notched arrow) on an unsubtracted image. (F) Complete resolution of left vertebral artery V4 segment stenosis following stent implantation. AICA: anterior inferior cerebellar artery; PICA: posterior inferior cerebellar artery.

**Figure 3:**
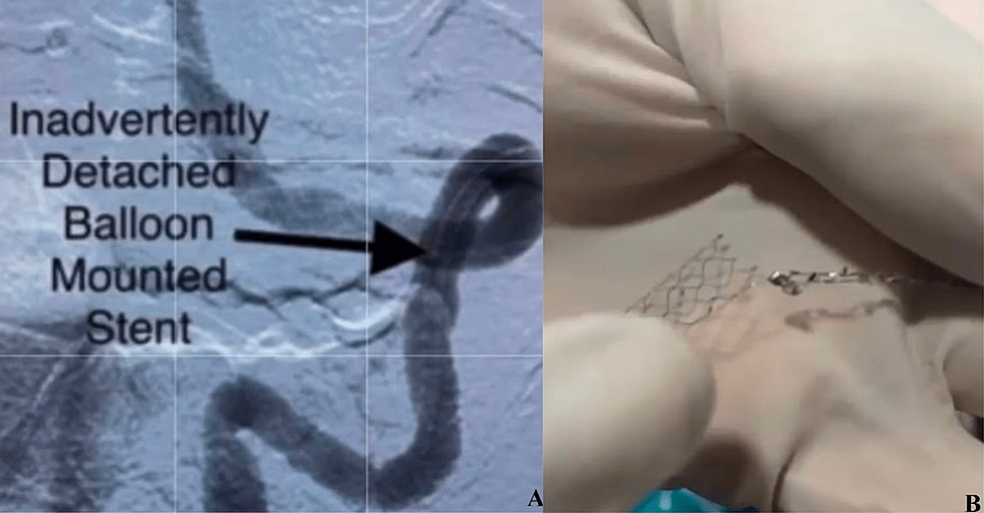
(A) Digital subtraction angiography showing the detached stent. (B) Stent fracture and displaced stent after retrieval.

Left radial access was obtained with a Terumo 6 Fr short sheath (Terumo Interventional Systems, New Jersey, USA) and a Neuron 053 guide catheter (Penumbra Inc, California, USA) was negotiated into the left vertebral artery. The guide catheter was positioned in the V3 segment and a Synchro 0.014 wire (Stryker Neurovascular, California, USA) was advanced across the stenosis distally into the left posterior cerebral artery (PCA). A 3.5 x 28 mm Promus PREMIER™ Select coronary stent (Boston Scientific, Massachusetts, USA) was used. There was difficulty in negotiating the stent through the vertebral artery V3 segment loop, and during the manipulation, the stent inadvertently got detached from its shaft (Figure 2). The Synchro 0.014 wire and the stent delivery system were removed. A control angiogram revealed the stent dangling in the vertebral artery V3 segment without causing any blood flow limitation (Figure 3A).

Stentectomy was decided upon and a Trevo Pro microcatheter (Stryker Neurovascular, California, USA) was negotiated alongside the detached stent in the vertebral artery V3 segment. A 4 x 40 Solitaire stent retriever device (Medtronic, Minnesota, USA) was deployed and the proximal end of the stent was captured (Figure 2D). The assembly of the stent retriever, stent, and Neuron 053 guide catheter were slowly withdrawn and removed through the radial sheath (Figure 3B and Video 1).

**Video 1:** Dislodged stent and its removal using stent retriever alone.

**Figure 4:**
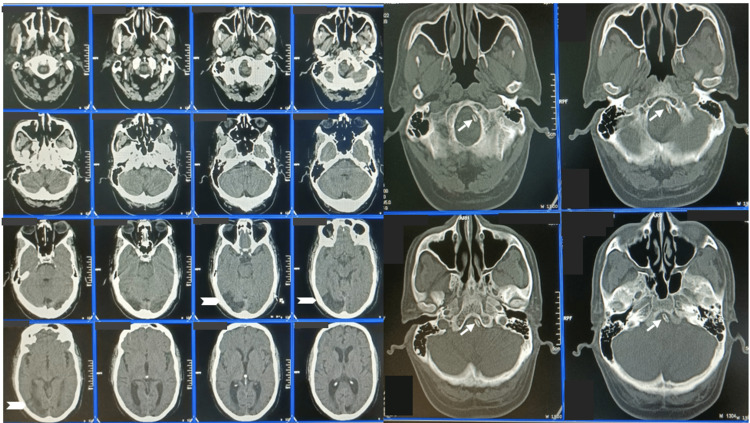
Post-procedural CT changes comparable with the pre-procedural MRI (notched arrow). The stent is seen in the bone window of post-procedure CT (arrow).

To have better proximal support, the Terumo 6 Fr short radial sheath was exchanged with a 6 Fr Cook shuttle sheath (Cook Medical, Indiana, USA) over a 0.035 GLIDEWIRE® Hydrophilic Coated Guidewire (Terumo Interventional Systems, New Jersey, USA). With the Cook shuttle sheath positioned in the left subclavian artery close to the vertebral artery ostium, a Stryker CAT® 5 Distal Access Catheter (Stryker Neurovascular, California, USA) was advanced into the left vertebral artery proximal V4 segment. A Synchro 0.014 wire was advanced across the stenosis and another 3.5 x 28 mm Promus PREMIER™ Select coronary stent could be tracked without much difficulty. Post-stenting control angiogram revealed complete resolution of stenosis and normal filling of all the distal branches. Throughout the procedure, the patient remained hemodynamically stable and was under conscious sedation. Post-procedural CT scan of the brain was unremarkable and the patient was discharged after three days of hospitalization (Figure 4). The patient was discharged on Ticagrelor 90 mg, Aspirin 75 mg, Rosuvastatin 10 mg, and corrective insulin therapy. He was referred to physical therapy for gait and balance training exercises. Currently, his ataxia has resolved completely and he is independent in all activities of his daily life.

## Discussion

Intracranial stenting has been advocated in the setting of severe intracranial atherosclerotic disease (ICAD) causing recurrent ischemic events despite aggressive medical management. Both self-expandable and balloon-mounted stents have been used for this purpose [6]. There can be some complications associated with either kind of stent. One such important complication is stent detachment and migration. Stent migration can be related to several factors: inserting while the balloon is exposed to negative pressure, inserting the stent through a previously deployed stent, advancing the stent through calcified, tortuous lesions, or during withdrawal of the stent to the guiding catheter while the proximal struts are damaged. The guiding catheter falling out as a result of tortuous anatomy can increase the risk rate of slip-off and stent migration [7]. Stent migration can be dangerous because it can either lead to traumatic perforation of the vessel or cause increased thrombogenicity and ischemia. Therefore, immediate management of stent migration is very crucial.

Stent detachment in this case possibly occurred because the guiding catheter buckled while negotiating the stent in the tortuous anatomical segment of the vertebral artery. Many bail-out techniques were described for coronary vasculature stent retrieval. The most common method is using a snare and capturing the stent between the loop and the delivery catheter tip [8]. The second most common method used is the small balloon technique, which employs a small balloon to be inflated distal to the stent enabling us to capture the stent and the balloon together as a unit into the guiding catheter [8]. Various devices such as loop snare, small balloons, baskets, grasping forceps, tip deflecting wires, pinching devices, oversize catheters, and balloon catheters are also successfully employed [8].

These techniques might not be the first choice for cerebral vasculature as these vessels have smaller diameters, and lack solid adherence to adjacent tissues. Successful stentectomy from cerebral vasculature has been reported using endovascular snares, merci retrieval systems, and alligator retrieval devices [3-5]. Some operators reported removing the stents surgically. Seong et al. described the co-axial snare technique, which emphasizes snaring the stent coaxially rather than simple snaring [9]. Parthasarathy et al. also described the loop and snare technique. In this technique, a microcatheter with a curved tip is first advanced through the distal part of the detached stent. The microwire is then navigated through the struts and back into the parent artery to form a loop. Now, a microcatheter is navigated over the microwire. A snare is advanced into the parent artery to snare the microwire. The microwire and snare complex is then pulled back to retrieve the stent [5]. They also described the deploy and engage technique, which essentially uses another similar-sized partially deployed solitaire stent to engage with the detached stent and resheating it to be removed as a whole [5]. Few case reports also reported using pull-back maneuvers using a balloon [8]. An experimental feasibility study was also performed using a combination of snare and stent retriever named as SOS (snare over stent retriever) technique. This technique uses a pre-deployed snare over a microcatheter to apply slight pressure while the misplaced stent is being removed with a stent retriever [10].

## Conclusions

Balloon-mounted stents are being used to treat intracranial atherosclerotic disease. Inadvertent stent detachment is a rare and infrequent complication during intracranial stenting procedures. Inadvertent stent detachment can be averted by having a stiffer proximal access support catheter, especially when using longer stents and negotiating through tortuous anatomy. Stent retrievers are easy to deploy and can be handy in extracting detached stents. We demonstrate a case where we successfully captured the detached stent and extracted it outside the body.
